# CTLA-4 affects expression of key cell cycle regulators of G0/G1 phase in neoplastic lymphocytes from patients with chronic lymphocytic leukaemia

**DOI:** 10.1007/s10238-015-0360-7

**Published:** 2015-05-24

**Authors:** Lidia Ciszak, Irena Frydecka, Dariusz Wolowiec, Aleksandra Szteblich, Agata Kosmaczewska

**Affiliations:** 1 Laboratory of Immunopathology, Department of Experimental Therapy, Ludwik Hirszfeld Institute of Immunology and Experimental Therapy, Polish Academy of Sciences, R. Weigla 12, 53-114 Wrocław, Poland; 2 Department and Clinic of Haematology, Blood Neoplasms, and Bone Marrow Transplantation, Wroclaw Medical University, L. Pasteura 4, 50-367 Wrocław, Poland

**Keywords:** Chronic lymphocytic leukaemia (CLL), CTLA-4 (CD152), Cyclin D2, Cyclin D3, p27^*KIP1*^

## Abstract

Previously, we showed that cytotoxic T-lymphocyte-associated antigen 4 (CTLA-4) is overexpressed in chronic lymphocytic leukaemia (CLL) and its expression is correlated with the expression of the major regulators of G1 phase progression: cyclins D2 and D3, and cyclin-dependent kinase inhibitory protein 1 (p27^*KIP1*^). In the present study, we blocked CTLA-4 on the surface of both CLL cells and normal B lymphocytes to investigate the impact of CTLA-4 on the expression of the mentioned G1 phase regulators. We found that in CLL patients and in healthy individuals, the median proportions of cyclin D2-positive cells as well as cyclin D3^+^ cells significantly decreased following CTLA-4 blockade. Moreover, CTLA-4 blockade led to an increase in the median frequencies of p27^*KIP1*^-positive cells, although this increase was marked only in CLL patients. Our study showed that CTLA-4 affects the expression of the key regulators of G1 phase progression in CLL cells as well as in normal B lymphocytes and may contribute to a better understanding of the role of CTLA-4 in the regulation of G1 phase progression.

## Introduction

Chronic lymphocytic leukaemia (CLL) is the most common form of leukaemia in adults in Western Europe and North America. It has been described as a progressive accumulation of malignant, morphologically mature CD19^+^CD5^+^ cells in the peripheral blood, bone marrow, and lymphoid organs [1–3]. The disease is characterised by a highly variable clinical presentation and evolution [4, 5]. Some patients exhibit stable disease over years and never require a therapeutic intervention, whereas others progress rapidly towards more advanced stages and succumb relatively soon despite aggressive treatment. Based on genetic, phenotypic, and molecular characteristics of CLL, several prognostic markers have emerged in the past decade [6, 7]. It has been well documented that the high-risk phenotype is typically associated with unmutated immunoglobulin heavy variable genes (IgV_H_) [8], high expression of the CD38 surface marker [9], or the ζ-chain-associated protein 70 kDa (ZAP-70) [10], as well as with chromosomal aberrations such as 17p (the site of tumour protein p53) or 11q23 deletions (the site of ataxia telangiectasia mutated ATM) [11]. Recently, it has been postulated that the difference in clinical course among CLL patients may, at least in part, be determined by the proliferative capacity of CLL cells [12, 13]. It is becoming increasingly evident that the cells located in the proliferation centres (PCs) of lymph nodes and bone marrow proliferate more than previously anticipated [13], and CLL patients with higher birth rates are much more likely to exhibit active or to develop progressive disease than those with lower birth rates [13]. Moreover, the proliferating cells in PCs represent the CLL proliferating reservoir that replenishes the downstream accumulation compartment.

In contrast to the proliferating cells in PCs, the vast majority of CLL cells circulating in the peripheral blood are arrested in the G0/early G1 phase of the cell cycle. The key regulators of G1 phase progression in human T and B lymphocytes are the D-type cyclins cyclin D2 and D3, and p27^*KIP1*^ (cyclin-dependent kinase inhibitory protein 1). The D-type cyclins positively regulate passage through the G1 phase by binding to and stimulating the activities of their catalytic partners, the cyclin-dependent kinases (CDKs) cdk4 and cdk6 [14–17], while p27^*KIP1*^ exerts an inhibitory effect on the kinases cdk4 and cdk6 or their complexes with cyclin D2 or cyclin D3 [17–19]. Moreover, one of the factors involved in regulation of cell cycle progression of T cells is cytotoxic T-lymphocyte-associated antigen 4 (CTLA-4; CD152) [20, 21]. It has been well documented that CTLA-4 prolongs the progression of T cells through the G1 phase by influencing the expression of the major regulators of this cell cycle phase [20, 21]. CTLA-4 up-regulates the expression of cyclin D2 and inhibits cyclin D3, cdk4, and cdk6 production in these cells. Furthermore, CTLA-4 affects the degradation of p27^*KIP1*^ protein and contributes to its earlier and stronger re-expression during the late stages of T cell activation [20, 21]. In contrast to the well-documented involvement of CTLA-4 in the regulation of cell cycle progression in T cells [20, 21], only limited information is known about the role of this protein in cell cycle progression in normal B cells and malignant B lymphocytes. Our previous study indicated that CTLA-4 is overexpressed in freshly drawn CLL cells and it may be involved in the regulation of G1 phase progression in these cells [22]. We found that CTLA-4 expression positively correlated with both cyclin D2 and p27^*KIP1*^ expression and negatively with cyclin D3 level. Moreover, CTLA-4 expression positively correlated with the percentage of leukaemic cells in G0/G1 phase. Here, we have extended our previous study to examine whether stimulation with DSP30, a CpG oligodeoxynucleotide (ODN), and rIL-2 influences CTLA-4 expression in CLL cells. The main aim of this study was to investigate whether the CTLA-4 molecule affects the expression of cell cycle regulators of G0/G1 phase. For that purpose, we blocked CTLA-4 on the surface of CLL cells using monoclonal anti-CTLA-4 antibodies to assess the expression of cyclins D2 and D3, and p27^*KIP1*^ protein. To the best of our knowledge, such studies are lacking so far.

## Materials and methods

### Patients and healthy donors

The study design was approved by the local Bioethical Committee at the Medical University of Wroclaw, Poland, and is in accordance with the Helsinki Declaration of 1975. All participants gave written informed consent after the purpose of the study was explained to them. Thirty-eight previously untreated CLL patients of the Clinic of Haematology, Blood Neoplasms, and Bone Marrow Transplantation, Wroclaw Medical University, Poland, were enrolled in this study. In each of them, the diagnosis was established according to generally accepted criteria including absolute peripheral blood lymphocytosis ≥5 × 10^9^/L and the co-expression of CD5, CD19, and CD23 antigens on malignant cells. The disease stages were determined according to the Rai classification. Clinical and laboratory features are presented in Table 1.

**Table 1:** Clinical characteristics of CLL patients

Characteristics	Value
Number of patients	38
Gender (female/male)	15/23
Age (years, mean and SD)	69.0 ± 11.2
Rai stage	
0	14
I	7
II	6
III	4
IV	7
WBC count (1 × 10^9^/l)	72.2 ± 98.4
Lymphocyte count (1 × 10^9^/l)	66.5 ± 96.8
Hb level (g/dl)	12.9 ± 1.9
Platelet count (1 × 10^9^/l)	135.2 ± 55.1
LDH (U/l)	206.8 ± 55.3
β2-microglobulin (mg/l)	3.3 ± 1.4

For age and clinical parameters, the mean values and standard deviation (SD) were presented

Leucocyte-enriched fractions of peripheral blood donated by 15 healthy volunteers matched for age and sex with the CLL patients were purchased from the Regional Centre of Blood Donation and Treatment in Wroclaw, Poland.

### Cell isolation and separation procedures

Peripheral blood mononuclear cells (PBMCs) were separated from heparinised freshly drawn peripheral venous blood of CLL patients and healthy controls by buoyant density-gradient centrifugation on Lymphoflot (Bio-Rad Medical Diagnostics GmbH, Dreieich, Germany) and washed three times in phosphate-buffered saline (PBS) (without Ca^2+^ and Mg^2+^). The PBMCs were suspended in 95 % foetal calf serum (CytoGen GmbH, Sinn, Germany) containing 5 % DMSO (Sigma-Aldrich, St. Gallen, Switzerland) and stored in liquid nitrogen until used.

CLL cells were isolated from PBMCs by negative selection using EasySep Human B Cell Enrichment Kit without CD43 Depletion (STEMCELL Technologies Inc, Vancouver, Canada) according to the manufacturer’s instructions. Following this separation procedure, more than 98 % of the resulting cell population was CD19^+^CD5^+^ as assessed by flow cytometry using anti-CD19 and anti-CD5 monoclonal antibodies (mAbs) (Becton–Dickinson, BD Biosciences, San Diego, USA). Normal B cells from healthy individuals were isolated from PBMCs by negative selection using EasySep Human B Cell Enrichment Kit (STEMCELL Technologies Inc, Vancouver, Canada) according to the manufacturer’s instructions, achieving above 98 % purity as assessed by flow cytometry using anti-CD19 mAb.

### Culture conditions

Purified normal CD19^+^ lymphocytes or CLL cells were suspended at 1 × 10^6^ cells/ml in RPMI-1640 medium (Gibco, Paisley, UK) supplemented with 10 % foetal calf serum (CytoGen GmbH, Sinn, Germany), 2 mmol/l l-glutamine and 50 μg/ml gentamycin (KRKA-Poland, Warsaw, Poland), and cultured using 24-well U-bottom culture plates (Nunc GmbH & Co. KG, Langenselbold, Germany) at 37 °C in a 5 % CO_2_ humidified atmosphere for 24 and 72 h either in medium alone or together with 1 µM DSP30 (5′-TCGTCGCTGTCTCCGCTTCTTCTTGCC-3′) (TIB MOLBIOL, Berlin, Germany) [23] and 100 U/ml rIL-2 (Eurocetus, Amsterdam, The Netherlands) [24]. For the blocking experiment, purified CLL cells and normal CD19^+^ lymphocytes were cultured with 1 µM DSP30 and 100 U/ml IL-2 with the blocking anti-CTLA-4 mAbs (50 µg/ml) (BD Pharmingen, BD Biosciences, San Diego, USA) [25] or control IgG2 (50 µg/ml) (BD Pharmingen, BD Biosciences, San Diego, USA).

### Immunostaining of CTLA-4 and cell cycle regulators of G0/G1 phase, and flow cytometric analysis

The expression of these molecules was studied in purified CLL cells and normal CD19^+^ lymphocytes before and after 24 and 72 h culture by a single immunostaining method.

Briefly, for detection of surface expression of the CTLA-4 molecule, the cells were washed twice in PBS (without Ca^2+^ and Mg^2+^), divided into tubes at a concentration of 5 × 10^5^ cells per tube and incubated with anti-CTLA-4 (CD152)/RPE mAbs (BD Pharmingen, BD Biosciences, San Diego, USA) for 30 min at 4 °C in the dark. Excess unbound antibodies were removed by two washes with PBS. Following these washes, the cells were resuspended in PBS and analysed by flow cytometry using a FACSCalibur flow cytometer (Becton–Dickinson, BD Biosciences, San Diego, USA). For determination of intracellular CTLA-4 expression, the cells were first fixed for 10 min at room temperature in 2 % paraformaldehyde (Fluka, Sigma-Aldrich, Buchs, Germany), washed in PBS, and incubated for 10 min at room temperature in BD Permeabilizing Solution 2 (Becton–Dickinson, BD Biosciences, San Diego, USA) according to the manufacturer’s instructions. Then, the cells were incubated with anti-CTLA-4 (CD152)/RPE mAbs for 30 min at 37 °C in the dark.

For detection of cyclins D2 and D3, and p27^*KIP1*^ protein, the cells were fixed, permeabilised, and stained with anti-cyclin D2/FITC, anti-p27^KIP1^/FITC mAbs (Santa Cruz Biotechnology, Inc, Heidelberg, Germany), and anti-cyclin D3/FITC mAb (BD Pharmingen, BD Biosciences, San Diego, USA) according to the manufacturer’s instructions.

Negative controls were always done by omitting the mAbs and by incubating the cells with mouse Ig of the same isotype as the mAbs conjugated with RPE or FITC. At least, 10,000 events per sample were analysed. The results were expressed as the proportion of CTLA-4-, cyclin D2-, cyclin D3-, or p27^*KIP1*^-positive cells. The CellQuest program was used for statistical analysis of the acquired data.

### Statistical analysis

Statistical analyses of the clinical data and laboratory findings were conducted using Statistica 10.0 or PQStat software. For clinical parameters, the mean values and standard deviation were calculated. For all other analysed variables, the median values and 25th and 75th interquartile range were calculated. All collected data were examined for normal distribution and for homogeneity of variances using the Shapiro–Wilk test and Levene’s test, respectively. If data were normally distributed and had homogeneous variances, the comparisons between the studied groups were performed using the Student *t* test for independent samples. If data were not normally distributed and/or had heterogeneous variances, the nonparametric Mann–Whitney *U* test was used. To test the effects of culture and CTLA-4 blockade on analysed variables, the repeated measures ANOVA and the Student *t* test for dependent samples were used. If data were not normally distributed and/or had heterogeneous variances, the Friedman ANOVA test followed by a post hoc test (Dunn test), and the nonparametric Wilcoxon signed-rank test were applied. The relationship between the clinical parameters and CTLA-4 expression was tested with Spearman’ rank correlation coefficient. In all analyses, differences were considered significant when *P* ≤ 0.05.

## Results

### Expression of CTLA-4 molecule and cell cycle regulators of G0/G1 phase in freshly drawn CLL cells and normal CD19^+^ lymphocytes

**Fig. 1:**
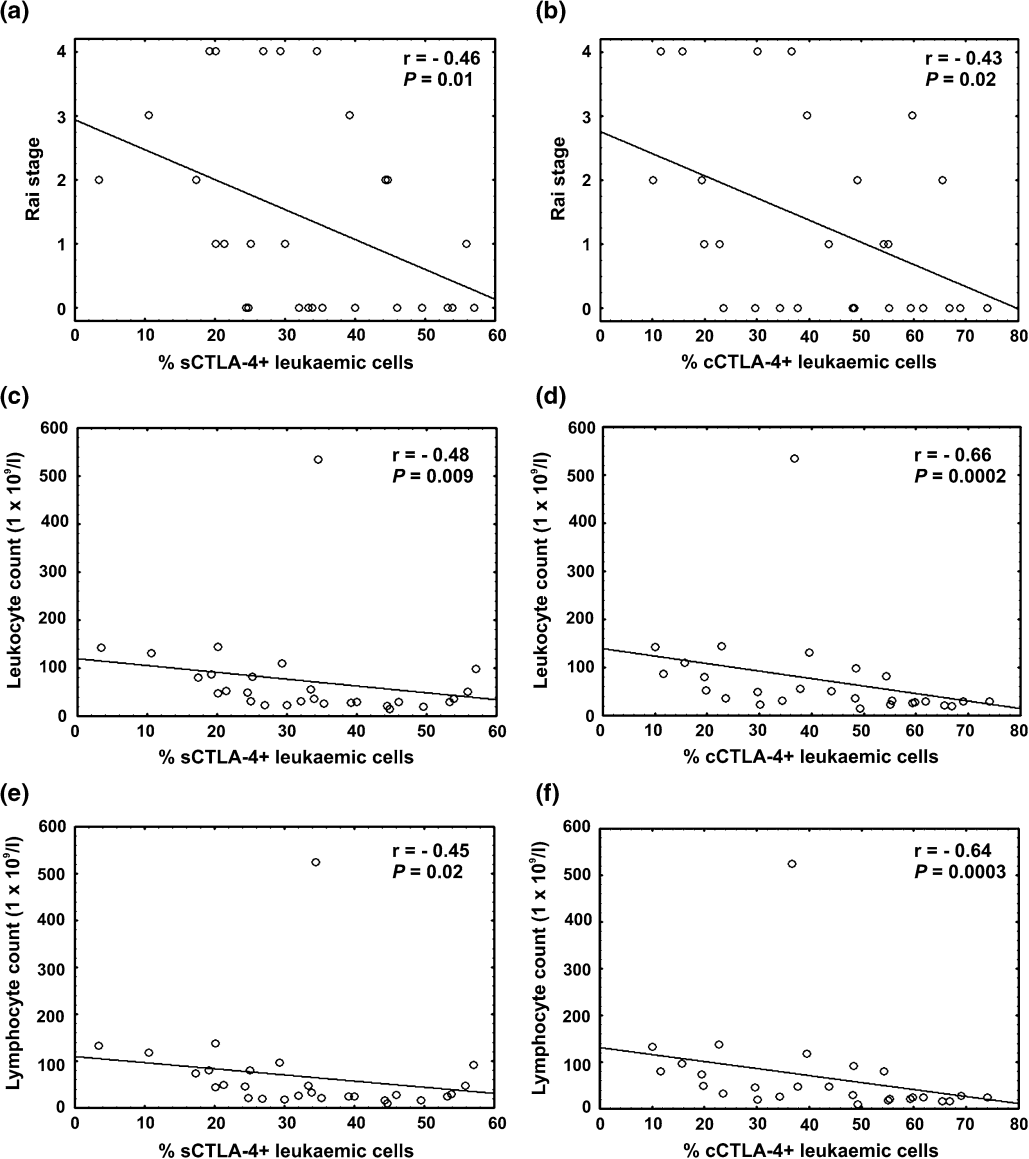
Correlation between CTLA-4 expression and clinical parameters. **a** Negative association between proportions of sCTLA-4-positive leukaemic cells and Rai stages. **b** Negative correlation between percentages of cCTLA-4-positive leukaemic cells and Rai stages. **c** Negative association between frequencies of sCTLA-4-positive leukaemic cells and leucocyte count. **d** Negative correlation between percentages of cCTLA-4-positive leukaemic cells and leucocyte count. **e** Negative association between proportions of sCTLA-4-positive leukaemic cells and lymphocyte count. **f** Negative correlation between frequencies of cCTLA-4-positive leukaemic cells and lymphocyte count

Since CTLA-4 is transiently expressed on the cell surface and is predominantly located in intracellular compartments due to constitutive internalisation from the plasma membrane [26], we determined both surface and intracellular expression of this molecule. Similarly, to our earlier study [22], we observed significantly higher median proportions of leukaemic cells co-expressing the CTLA-4 molecule on the surface (sCTLA-4) as well as in cytoplasmic compartments (cCTLA-4) in CLL patients compared to the median percentages of the corresponding cells in healthy individuals (Table 2). Moreover, as we previously showed [22], the frequencies of both the sCTLA-4-positive and cCTLA-4-positive CLL cells were very variable in the studied patients, ranging from 3.5 to 57.1 % and from 10.1 to 74.1 %, respectively. Furthermore, we found negative correlations between sCTLA-4 as well as cCTLA-4 expression in leukaemic cells and the following clinical parameters: Rai stage (*r* = −0.46, *P* = 0.01 and *r* = −0.43, *P* = 0.02), leucocyte count (*r* = −0.48, *P* = 0.009 and *r* = −0.66, *P* = 0.0002), and lymphocyte count (*r* = −0.45, *P* = 0.02 and *r* = −0.64, *P* = 0.0003) (Fig. 1).

**Table 2:** **M**edian proportions and 25th–75th interquartile range of CLL cells and normal B lymphocytes co-expressing CTLA-4 molecule

Groups	Unstimulated	24-h culture		72-h culture	
		Medium alone	DSP30+rIL-2	Medium alone	DSP30+rIL-2
*% sCTLA-4-positive cells*					
CLL patients (*n* = 38)	32.8 (22.9–44.6)	28.6 (22.8–37.5)	25.8 (22.0–29.1)	18.2 (13.9–25.0)	17.7 (12.9–26.3)
Controls (*n* = 15)	18.8 (17.9–20.1)	17.9 (11.4–24.9)	20.9 (13.2–28.5)	11.8 (5.6–14.9)	14.8 (11.1–24.5)
CLL patients versus controls	*P* = 0.001	*P* = 0.03	NS	NS	NS
*% cCTLA-4-positive cells*					
CLL patients (*n* = 38)	43.8 (23.6–59.4)	51.0 (42.7–59.1)	52.1 (42.3–60.3)	42.4 (28.8–51.8)	44.7 (36.4–60.2)
Controls (*n* = 15)	25.7 (8.7–33.1)	30.4 (23.0–37.3)	26.2 (21.3–40.4)	14.1 (9.6–21.3)	19.1 (4.5–27.0)
CLL patients versus controls	*P* = 0.004	*P* = 0.002	*P* = 0.0005	*P* = 0.0008	*P* = 0.000009

As regards cell cycle regulators of G0/G1 phase, we observed significantly higher median proportions of both cyclin D2-positive and p27^*KIP1*^-positive cells in the CLL group compared to the median percentages of the corresponding cells in healthy volunteers (Table 3). In contrast, the median frequency of cyclin D3^+^ cells in CLL patients was markedly lower compared to healthy controls (Table 3).

**Table 3:** Median proportions and 25th–75th interquartile range of CLL cells and normal B lymphocytes co-expressing cyclin D2, cyclin D3, and p27^*KIP1*^

Groups	Unstimulated	24-h culture		72-h culture	
		Medium alone	DSP30+rIL-2	Medium alone	DSP30+rIL-2
*% cyclin D2-positive cells*					
CLL patients (*n* = 38)	37.7 (26.5–52.9)	38.1 (16.4–52.4)	31.9 (13.4–52.5)	0.0 (0.0–23.5)	3.2 (0.0–10.5)
Controls (*n* = 15)	0.0 (0.0–14.2)	9.9 (7.3–14.2)	64.6 (43.2–75.3)	1.8 (0.0–8.1)	0.9 (0.0–36.6)
CLL patients versus controls	*P* = 0.004	*P* = 0.009	NS	NS	NS
*% cyclin D3-positive cells*					
CLL patients (*n* = 38)	53.2 (44.2–61.8)	51.5 (44.8–61.0)	54.9 (48.3–65.7)	35.8 (30.2–48.5)	38.5 (26.5–47.8)
Controls (*n* = 15)	64.4 (56.4–84.5)	65.5 (57.7–66.9)	77.3 (76.3–84.5)	51.9 (41.1–62.6)	56.9 (51.7–65.7)
CLL patients versus controls	*P* = 0.01	*P* = 0.01	*P* = 0.00001	NS	*P* = 0.0004
*% p27* ^*KIP1*^ *-positive cells*					
CLL patients (*n* = 38)	81.4 (69.2–87.3)	85.5 (75.1–91.3)	68.9 (46.1–80.4)	84.5 (7.4–91.2)	51.2 (37.9–66.5)
Controls (*n* = 15)	51.5 (28.4–65.4)	58.5 (21.1–77.4)	27.7 (7.9–58.5)	32.5 (16.0–40.2)	23.7 (7.4–36.0)
CLL patients versus controls	*P* = 0.00007	*P* = 0.0002	*P* = 0.0009	*P* = 0.000001	*P* = 0.0001

### Expression of CTLA-4 molecule in DSP30+rIL-2-stimulated CLL lymphocytes and normal CD19^+^ cells

**Fig. 2:**
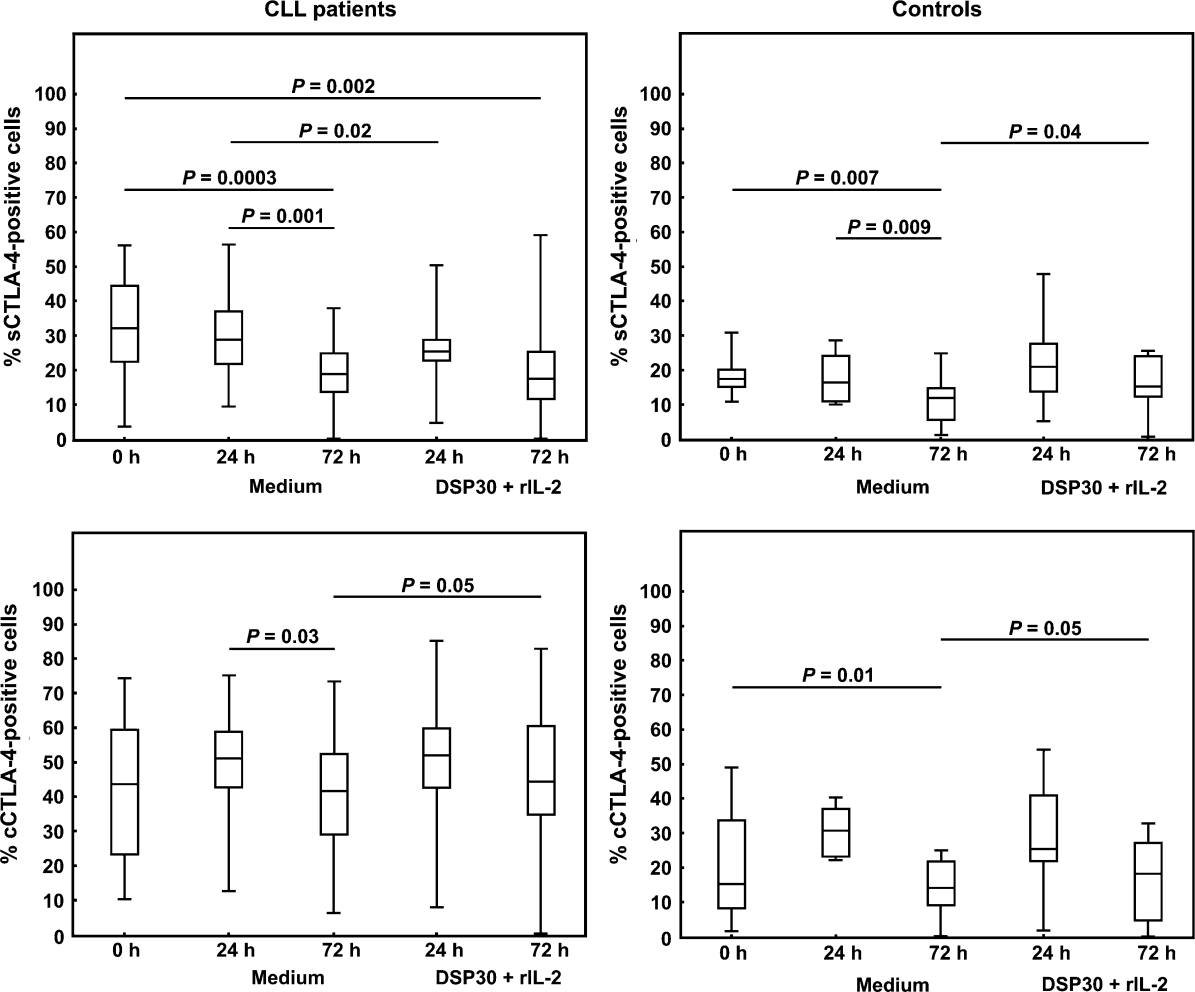
Surface and intracellular expression of CTLA-4 in leukaemic cells and normal B lymphocytes before and after cell culture. *Boxes* and *whiskers* 25th–75th interquartile range and minimum–maximum, respectively; the median is the *central line* in each *box*

**Fig. 3:**
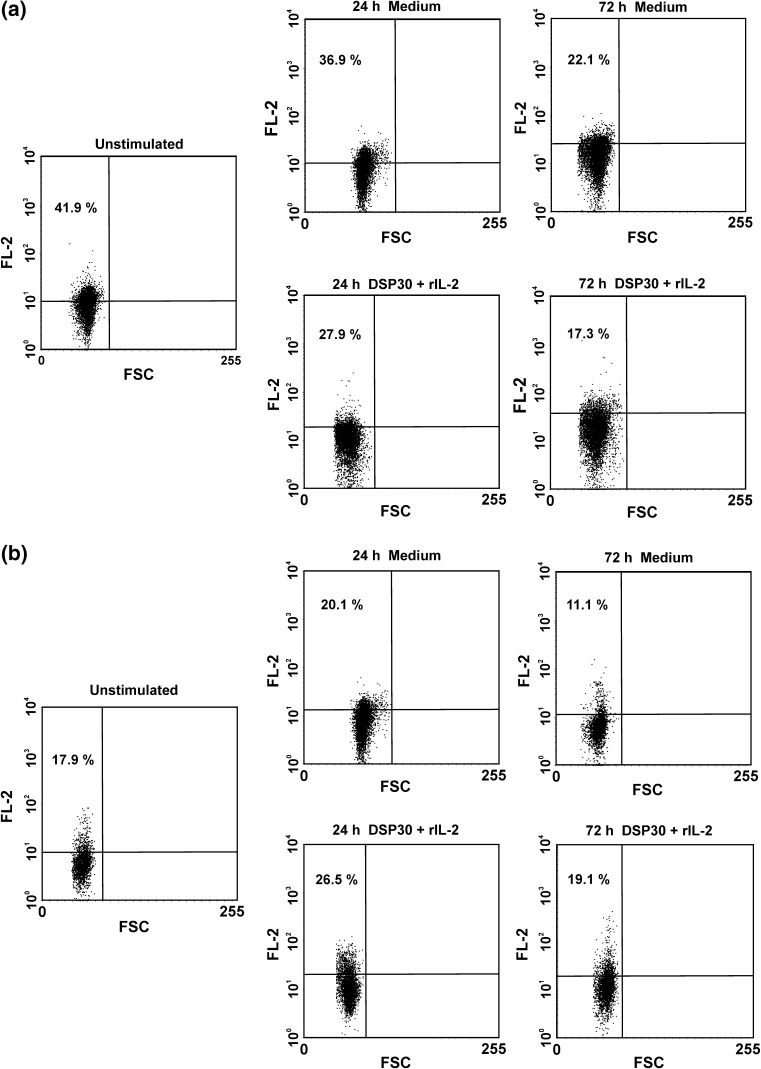
Representative examples of flow cytometric analyses of surface expression of CTLA-4 (sCTLA-4) on leukaemic cells (**a**) and normal B lymphocytes (**b**) before and after cell culture in medium alone and under stimulating conditions (DSP30+rIL-2). *Numbers* on *dot plots* represent the percentage of leukaemic or normal B lymphocytes expressing CTLA-4 on the cell surface

We found a significant impact of the cell culture in medium alone as well as ex vivo stimulation with DSP30+rIL-2 on the surface and intracellular expression of the CTLA-4 molecule in both CLL patients and healthy individuals. As regards surface expression, in CLL patients as well as in healthy individuals, a marked decrease in the median proportions of sCTLA-4-positive cells after 72 h of cell culture in medium alone was found (Table 2; Figs. 2, 3). Consequently, the median percentage of sCTLA-4^+^ cells in CLL patients remained higher than in healthy volunteers only after 24 h of culture (Table 2). In CLL patients, ex vivo stimulation led to a significant decrease in the median percentage of sCTLA-4^+^ cells after 24 h of culture (Table 2; Figs. 2, 3). In contrast, in healthy volunteers, stimulation with DSP30+rIL-2 resulted in an increase in the median frequencies of sCTLA-4-positive lymphocytes compared to the control culture, although the differences were statistically significant only after 72 h of culture (Table 2; Figs. 2, 3). Moreover, no significant differences in the median proportions of sCTLA-4^+^ cells between CLL patients and healthy individuals following ex vivo stimulation were found (Table 2).

**Fig. 4:**
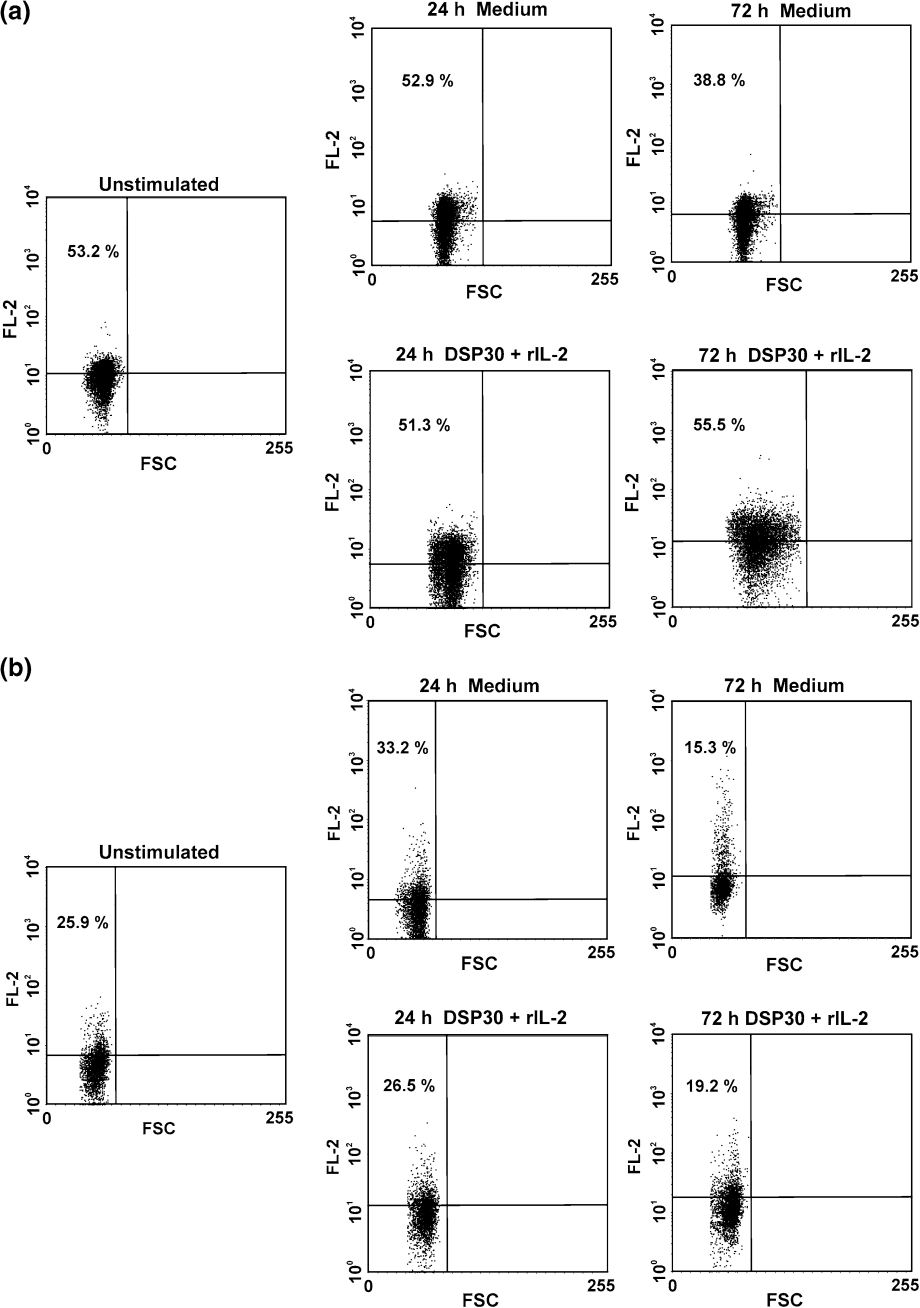
Representative examples of flow cytometric analyses of intracellular expression of CTLA-4 (cCTLA-4) in leukaemic cells (**a**) and normal B lymphocytes (**b**) before and after cell culture in medium alone and under stimulating conditions (DSP30+rIL-2). *Numbers* on *dot plots* represent the percentage of leukaemic or normal B lymphocytes expressing CTLA-4 in cytoplasmic compartments

As regards cCTLA-4 expression, in CLL patients as well as in healthy individuals, a significant decrease in the median proportions of cCTLA-4-positive cells after 72 h of cell culture in medium alone was found (Table 2; Figs. 2, 4). Moreover, after 72 h of ex vivo stimulation, the median percentages of cCTLA-4^+^ cells in both studied groups were significantly higher compared to the control culture (Table 2; Figs. 2, 4). Of note, the median proportions of cCTLA-4-positive cells in CLL patients remained higher than in healthy controls at each time point tested (Table 2).

### Expression of cell cycle regulators of G0/G1 phase in DSP30+rIL-2-stimulated CLL lymphocytes and normal CD19^+^ cells

**Fig. 5:**
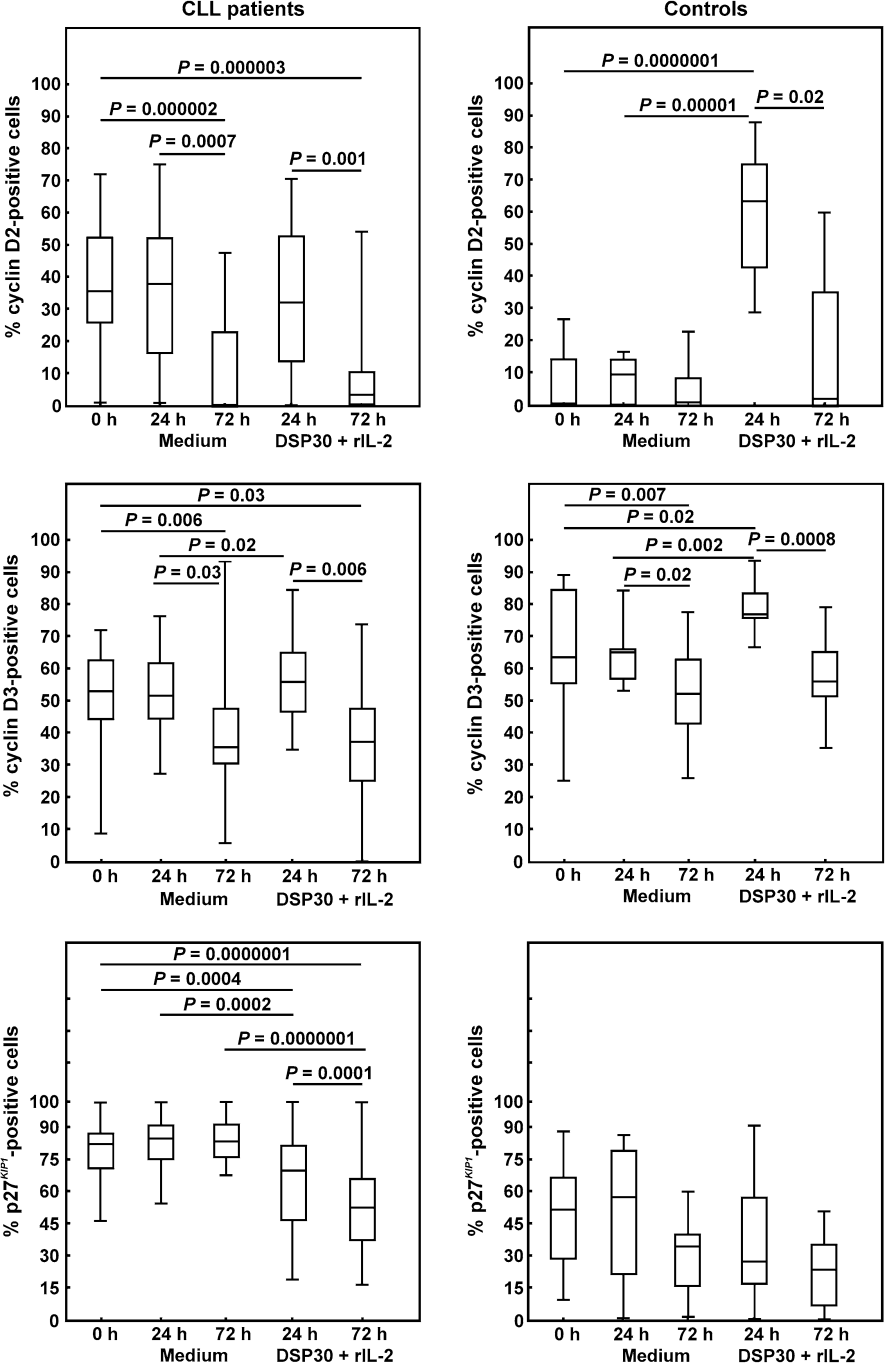
Cyclins D2 and D3, and p27^*KIP1*^ expression in leukaemic cells and normal B lymphocytes before and after cell culture. *Boxes* and *whiskers* 25th–75th interquartile range and minimum–maximum, respectively; the median is the *central line* in each *box*

Simultaneously with CTLA-4 expression, we analysed the expression of cell cycle regulators of G0/G1 phase. As regards cyclin D2 expression, culture in medium alone resulted in a marked decrease in the median percentage of cyclin D2-positive cells after 72 h of culture in CLL patients (Table 3; Fig. 5). In contrast, in healthy individuals, the median proportions of CD19^+^ cyclin D2^+^ lymphocytes maintained stable during the control culture (Fig. 5). In consequence, the median frequency of cyclin D2-positive cells in CLL patients remained significantly higher than in healthy controls only after 24 h of control culture (Table 3). Moreover, in CLL patients, ex vivo stimulation led to a significant decline in the median proportion of the cyclin D2^+^ cells after 72 h of cell culture compared to the value after 24 h of culture (Fig. 5). In contrast, in healthy individuals, after 24 h of ex vivo stimulation, we observed a marked increase in the median percentage of CD19^+^ cyclin D2^+^ cells (Fig. 5). Moreover, after 72 h of ex vivo stimulation, the median proportion of cyclin D2-positive cells markedly decreased compared to the value after 24 h of culture (Fig. 5). Furthermore, no significant differences in the median percentages of cyclin D2^+^ cells between CLL patients and healthy individuals following ex vivo stimulation were found (Table 3).

As regards cyclin D3 expression, culture in medium alone resulted in a marked decrease in the median percentage of cyclin D3-positive cells after 72 h in CLL patients as well as in healthy volunteers (Table 3; Fig. 5). Moreover, the median percentage of cyclin D3^+^ cells in CLL patients remained markedly lower than in healthy volunteers only after 24 h of control culture (Table 3). In both studied groups, after 24 h of ex vivo stimulation, we observed a significant increase in the median proportions of cyclin D3^+^ cells (Fig. 5). Moreover, in CLL patients as well as in healthy individuals, the median frequencies of cyclin D3-positive cells decreased markedly after 72 h of stimulating culture compared to the corresponding cells after 24 h of ex vivo stimulation (Fig. 5). Furthermore, in CLL patients, the median proportions of cyclin D3-positive cells were markedly lower compared with healthy volunteers under stimulation conditions.

As regards p27^*KIP1*^ expression, in both studied groups, no significant impact of the culture in medium alone on the expression of this protein was found (Table 3; Fig. 5). In CLL patients, ex vivo stimulation led to a gradual decrease in the median frequencies of p27^*KIP1*^-positive cells with the minimum values after 72 h of culture. Likewise, in healthy individuals, the median percentages of CD19^+^p27^*KIP1*+^ cells gradually declined under stimulation conditions and reached a minimum level after 72 h, but these decreases were not statistically significant. Moreover, the median frequencies of p27^*KIP1*^-positive cells in CLL patients remained markedly higher compared with healthy volunteers at each time point tested (Table 3).

### Effect of CTLA-4 blockade on expression of cell cycle regulators of G0/G1 phase in CLL lymphocytes and normal CD19^+^ cells

**Fig. 6:**
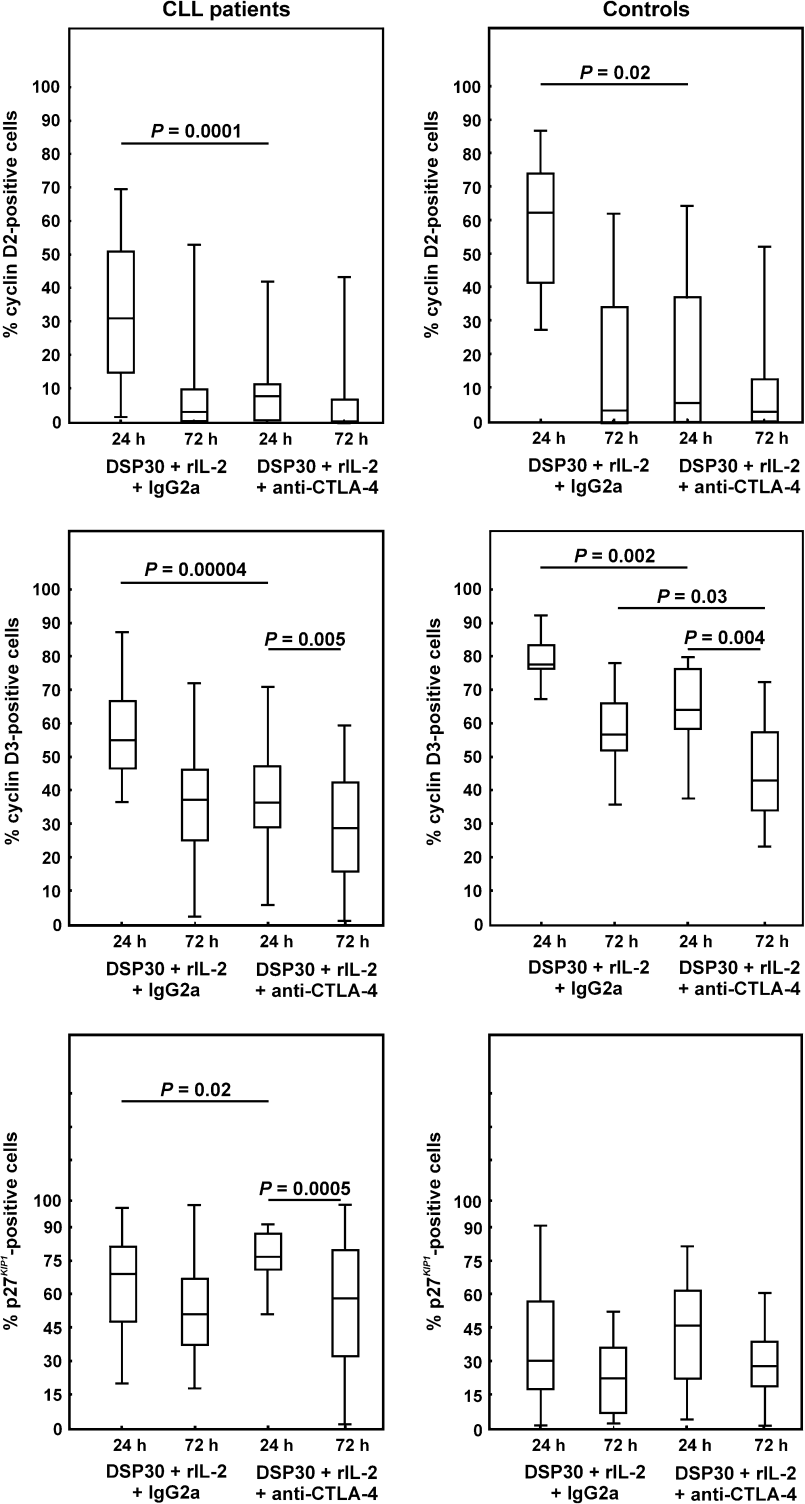
Effect of CTLA-4 blockade on the expression of cyclins D2 and D3, and p27^*KIP1*^ in leukaemic cells and normal B lymphocytes. *Boxes* and *whiskers* 25th–75th interquartile range and minimum–maximum, respectively; the median is the *central line* in each *box*

**Fig. 7:**
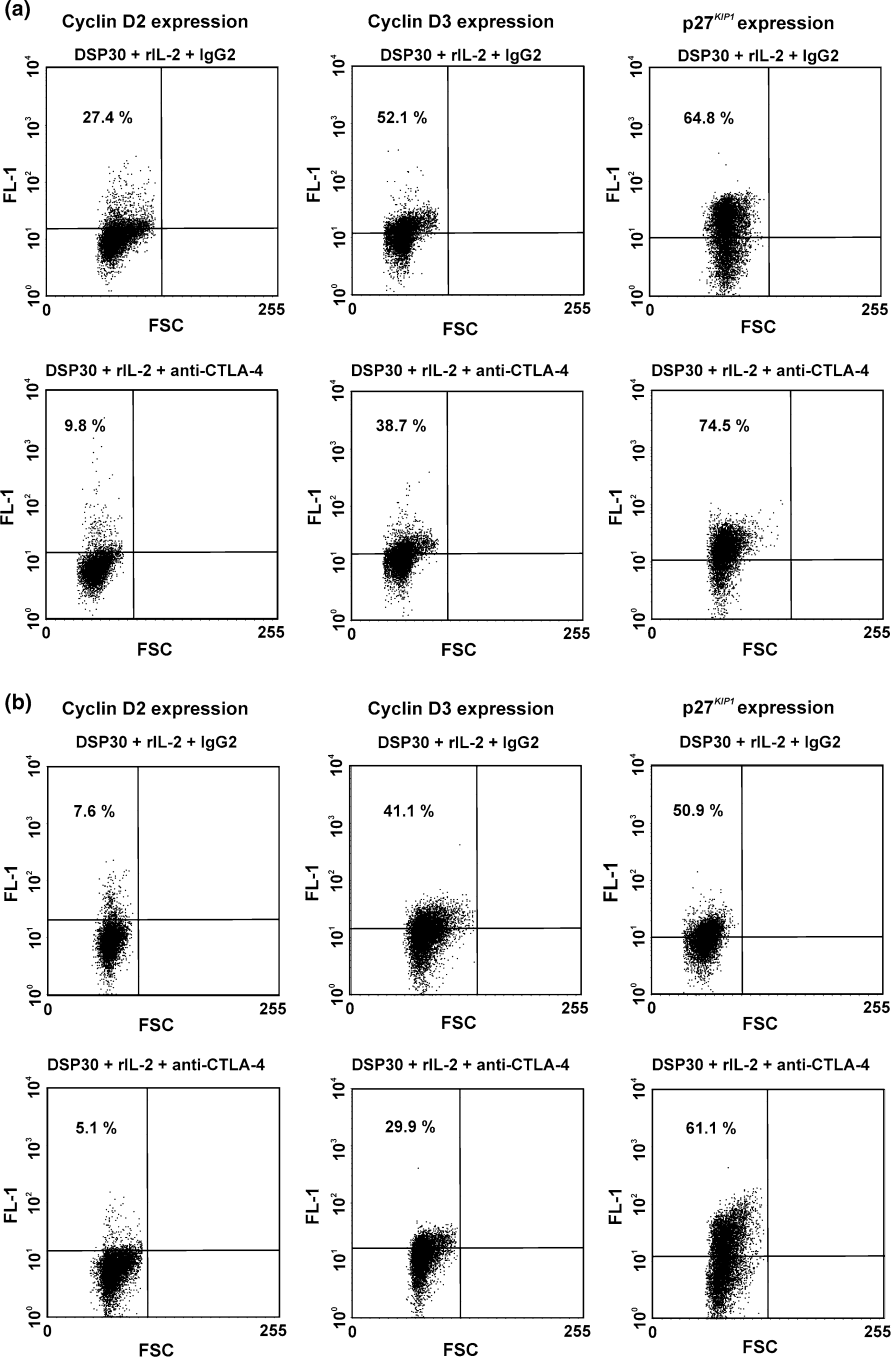
Representative examples of flow cytometric analysis of the effect of CTLA-4 blockade on expression of G0/G1 phase regulators in leukaemic cells after 24 h (**a**) and 72 h (**b**) of blocking culture. *Numbers* on *dot plots* represent the percentage of leukaemic cells expressing cyclin D2, cyclin D3 or p27^*KIP1*^

**Fig. 8:**
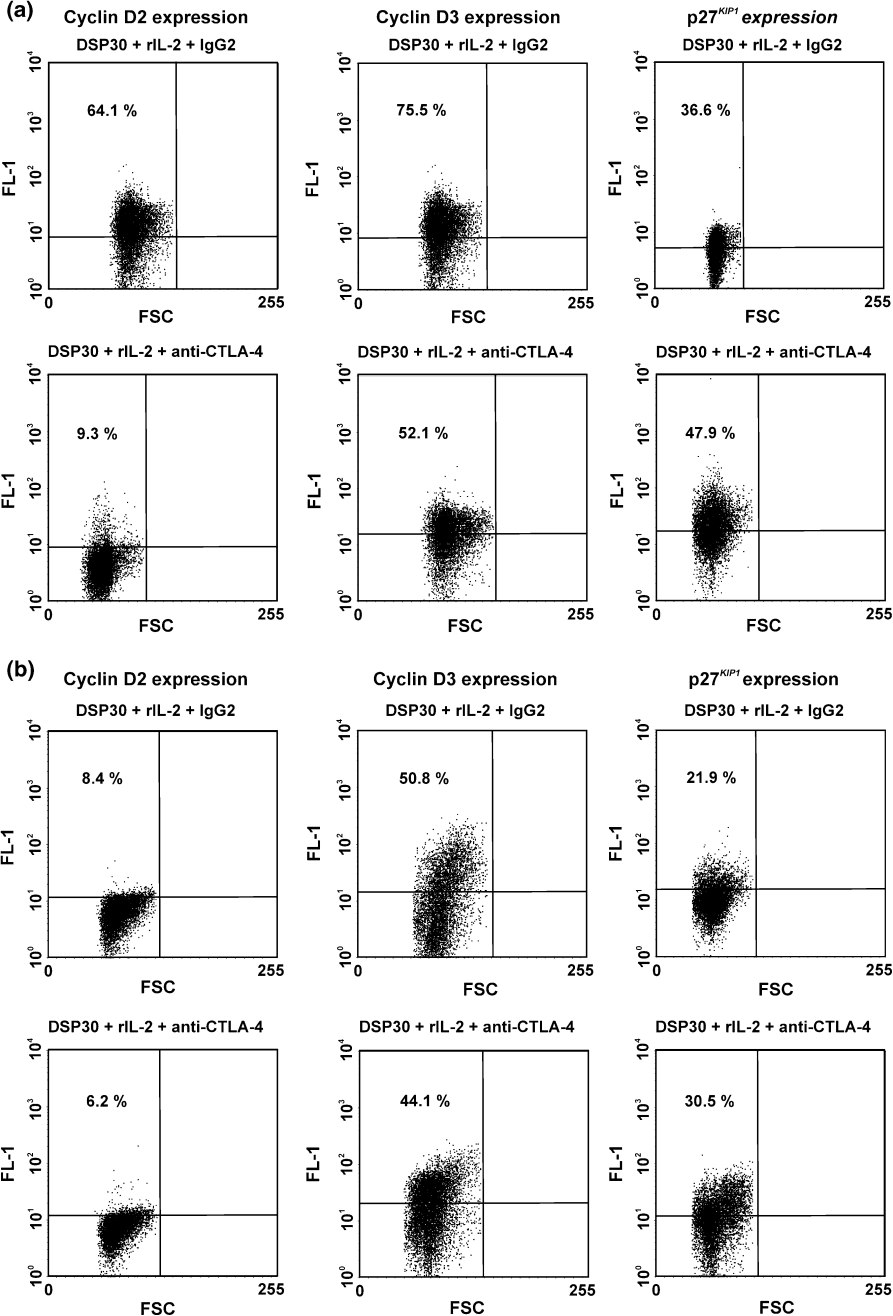
Representative examples of flow cytometric analysis of the effect of CTLA-4 blockade on expression of G0/G1 phase regulators in normal B lymphocytes after 24 h (**a**) and 72 h (**b**) of blocking culture. *Numbers* on *dot plots* represent the percentage of normal B lymphocytes expressing cyclin D2, cyclin D3 or p27^*KIP1*^

We next examined whether blocking CTLA-4 on the cell surface would affect the expression of the key regulators of G0/G1 phase. We observed a significant impact of CTLA-4 blockade on the expression of cell cycle regulators of G0/G1 phase in CLL patients as well as in healthy individuals. As regards cyclin D2, in both studied groups, we observed a marked decrease in the median proportions of cyclin D2-positive cells after 24 h of blocking culture (Figs. 6, 7, 8). Moreover, no significant differences in the median percentages of cyclin D2^+^ cells between CLL patients and healthy individuals were found.

As regards cyclin D3 expression, after 24 h of blocking culture, in CLL patients, we observed a marked decrease in the median proportions of cyclin D3-positive cells (Figs. 6, 7). In healthy volunteers, CTLA-4 blockade led to a decrease in CD19^+^ cyclin D3^+^ cells after 24 and 72 h of culture (Figs. 6, 8). Moreover, in CLL patients and in healthy individuals, after 72 h of CTLA-4 blocking culture, the median frequencies of cyclin D3-positive cells were markedly lower compared to corresponding cells after 24 h of culture (Figs. 6, 7, 8). Furthermore, the median frequencies of cyclin D3-positive leukaemic cells in CLL patients were markedly lower compared with healthy volunteers at each time point tested (*P* = 0.00002 and 0.005 after 24 and 72 h of blocking culture, respectively).

As regards p27^*KIP1*^ expression, in CLL patients, CTLA-4 blockade resulted in an increase in the median proportions of p27^*KIP1*^-positive cells compared to corresponding cells under stimulating conditions, although this increase was statistically significant only after 24 h of blocking culture (Figs. 6, 7). Moreover, in CLL patients, after 72 h of CTLA-4 blocking culture, the median frequency of p27^*KIP1*+^ cells was markedly lower compared to corresponding cells after 24 h of culture (Figs. 6, 7). In healthy individuals, an increase in the median proportions of p27^*KIP1*^-positive lymphocytes following 24 and 72 h of blocking culture was observed, although this increase was not statistically significant (Figs. 6, 8). Moreover, the median frequency of p27^*KIP1*^-positive cells in CLL patients remained markedly higher compared with healthy individuals at each time point tested (*P* = 0.000005 and 0.003 after 24 and 72 h of blocking culture, respectively).

## Discussion

In the present study, we confirmed our earlier observations [22] that both the surface and cytoplasmic expression of the CTLA-4 molecule is overexpressed in freshly isolated peripheral blood CLL cells. This finding raises a question about the mechanisms underlying the elevated levels of CTLA-4 expression in CLL cells. Since CTLA-4 expression is transiently inducible during T and B activation [27, 28] and it is considered to be an activation marker, the higher expression of CTLA-4 in CLL cells may reflect the systemic activation status in the periphery in CLL patients. It has been well documented that CLL cells receive many growth-promoting signals [29–31], which are mediated by receptors constitutively expressed on the subset of neoplastic B cells [32, 33]. In addition, literature data confirm that CLL cells bear the phenotype of activated B cells based on overexpression of the classical activation markers CD23, CD25, CD69 and CD71, and under-expression of CD22, Fcγ IIb, CD79b, and immunoglobulin D, which are down-regulated by cell triggering and activation [34]. The suggestion that CLL cells may be in a partial state of activation also results from the observation of cyclin D2 expression, which is induced during cell stimulation by growth factors in early G0 phase. Accordingly, in the current study, we confirmed our previous observations [22, 35, 36] that the median proportion of freshly isolated CLL cells co-expressing cyclin D2 was markedly higher than that observed in normal B lymphocytes. Furthermore, based on the fact that *CTLA*-*4* gene polymorphisms may influence the CTLA-4 expression level in CLL cells [22, 37], we cannot exclude that the strong divergence of CTLA-4 expression observed among the patients might result, at least in part, from the presence of specific alleles predisposing to overexpression of the CTLA-4 molecule.

We also found a negative correlation between CTLA-4 expression in CLL cells and the clinical parameters such as Rai stage and leucocyte as well as lymphocyte count. This finding is consistent with the study of Joshi et al. [38] showing that the level of *CTLA*-*4* gene expression in CLL cells predicts clinical outcome of CLL patients; lower expression of CTLA-4 is associated with significantly shorter time to treatment and poor prognosis compared with high CTLA-4 expression associated with good clinical outcome.

In the present study, we showed for the first time the impact of ex vivo specific stimulation of CLL cells with DSP30 (CpG ODN)+rIL-2 on CTLA-4 expression. An assessment of CTLA-4 expression under this stimulation condition seems to have clinical relevance in the context of the discussion about using CpG ODNs as therapeutic agents in CLL patients [39]. We found that ex vivo stimulation led to a significant decrease in the median proportion of CLL cells expressing the CTLA-4 molecule on their surface, which became comparable to the value of the corresponding cells in healthy individuals. In contrast, the intracellular expression of this protein in leukaemic cells markedly increased after 72 h of cell culture in the presence of DSP30 and rIL-2 and remained significantly higher than in healthy volunteers. This observation seems to suggest that the down-regulation of sCTLA-4 expression upon stimulation may result from disturbed recycling of the CTLA-4 molecule to the cell surface in CLL. Moreover, as higher expression of the *CTLA*-*4* gene is associated with good clinical outcome [38], application of CpG ODN that reduces the surface expression of CTLA-4 in leukaemic cells as a therapeutic agent may not be beneficial for CLL patients.

Literature data show that ex vivo stimulation with CpG ODN results in down-regulation of p27^*KIP1*^ expression and up-regulated expression of cyclins D2 and D3 in CLL cells and normal B lymphocytes [40–43]. In the present study, we also observed the down-regulation of p27^*KIP1*^ expression following ex vivo stimulation in CLL patients as well as in healthy volunteers. Of note, in spite of the decrease in p27^*KIP1*^ expression in both studied groups, the median frequencies of p27^*KIP1*^-positive cells in CLL patients remained significantly higher compared with healthy individuals at each time point tested. It is noteworthy that high expression of this protein is associated with poorer outcome of CLL [44–46]. As regards cyclin D2, in contrast to others [40–42], after 24 h of stimulating culture, we did not observe up-regulation of cyclin D2 expression in CLL cells. This discrepancy might result from the different experimental procedures used. We estimated the expression of these proteins as the proportions of CLL cells co-expressing several proteins using flow cytometry, whereas the authors of the contradictory reports examined the protein expression by western blot analysis. Due to cyclin D2 being overexpressed in freshly drawn CLL cells compared to normal B lymphocytes [22, 35, 36, 47] and maintained at similar high levels in CLL cells after 24 h of ex vivo stimulation, the median percentage of cyclin D2-positive cells in CLL patients was comparable to the corresponding cells in healthy individuals, in which up-regulation of cyclin D2 expression was observed. Moreover, in agreement with literature data [40–43], we found a significant increase in the median percentages of cyclin D3-positive cells after 24 h of ex vivo stimulation in CLL patients as well as in healthy volunteers. Of note, the median proportions of CLL cells co-expressing cyclin D3 remained markedly lower at each time point tested compared to corresponding cells in healthy individuals. To elucidate this phenomenon, further studies are necessary.

One of the main aims of this study was to examine whether the CTLA-4 molecule is able to influence the expression of the key regulators of G0/G1 phase in CLL cells as well as normal B lymphocytes. Our previous data [22] indicated that expression of the CTLA-4 molecule in CLL cells correlated with the expression of the G0/G1 phase regulators cyclins D2 and D3, and p27^*KIP1*^, suggesting the impact of CTLA-4 on prolonging the G1 phase in leukaemic lymphocytes. In the present study, we examined the expression of the mentioned cell cycle regulators following blockade of CTLA-4 on the surface of CLL cells as well as normal B lymphocytes. Blocking CTLA-4 on the surface of the studied cells is a useful method in research to elucidate the function of this protein. We found that CTLA-4 blockade did affect the expression of studied regulators of G0/G1 phase. In particular, we observed for the first time a significant decrease in the median proportions of both cyclin D2- and cyclin D3-positive cells in CLL cells as well as in normal B lymphocytes following 24 h of blocking culture. Moreover, we noted that CTLA-4 blockade led to an increase in the median proportions of p27^*KIP1*^-positive cells after 24 h of culture in CLL cells as well as in normal B lymphocytes, although this increase was statistically significant only in the group of CLL patients. What are the possible mechanisms responsible for this phenomenon? It has been shown that the expression of D-type cyclins and p27^*KIP1*^ protein stability is controlled by PI3 K (phosphatidylinositol 3-kinase)-related pathways [48, 49]. Activation of PI3 K-mediated pathways leads to transcription of D-type cyclins and to reduction of the p27^*KIP1*^ protein level [48, 49]. In some experimental systems, it has been shown that CTLA-4 engagement on T cells activates PI3K [50]. As CTLA-4 blockade has an opposite effect, it seems highly probable that CTLA-4 blockade may lead to inactivation of PI3K-related pathways. Based on this hypothesis, we can speculate that blockade of CTLA-4 on CLL cells as well as on normal B lymphocytes may lead to inactivation of PI3K-related pathways, resulting in decreased expression of cyclins D2 and D3, and accumulation of p27^*KIP1*^ protein, in consequence. Further studies are required to confirm a direct relationship between CTLA-4, PI3K-related pathways, and the expression of the mentioned cell cycle regulators of G0/G1 phase.

Furthermore, an interesting finding was that the median proportions of cyclin D2-positive cells in CLL patients and healthy volunteers were comparable following CTLA-4 blockade, whereas the median percentages of cyclin D3^+^ and of p27^*KIP1*+^ leukaemic cells remained lower and higher, respectively, than in healthy individuals. Further investigations are needed to explain this phenomenon.

Extending our previous study, we have demonstrated by blocking experiments that the CTLA-4 molecule influences the expression of the key regulators of G0/G1 phase—cyclins D2 and D3, and p27^*KIP1*^—in both malignant and normal B lymphocytes. We also confirmed the recent observation that CTLA-4 expression in peripheral blood CLL cells negatively correlates with disease progression, suggesting its clinical relevance as a prognostic marker. Since CTLA-4, cyclin D2 and p27^*KIP1*^ seem to have prognostic significance in CLL, further studies are necessary to elucidate the molecular mechanisms of the relationship between these proteins.

## References

[1] Chiorazzi N, Rai KR, Ferrarini M (2005). Chronic lymphocytic leukemia. N Engl J Med.

[2] Hallek M, Cheson BD, Catovsky D, Caligaris-Cappio F, Dighiero G, Dohner H, Hillmen P, Keating MJ, Montserrat E, Rai KR, Kipps TJ (2008). Guidelines for the diagnosis and treatment of chronic lymphocytic leukemia: a report from the International Workshop on chronic lymphocytic leukemia updating the National Cancer Institute-Working Group 1996 guidelines. Blood.

[3] Caligaris-Cappio F, Ghia P (2007). The normal counterpart to the chronic lymphocytic leukemia B cell. Best Pract Res Clin Haematol.

[4] Zenz T, Dohner H, Stilgenbauer S (2007). Genetics and risk-stratified approach to therapy in chronic lymphocytic leukemia. Best Pract Res Clin Haematol.

[5] Hamblin TJ, Oscier DG (1997). Chronic lymphocytic leukaemia: the nature of the leukaemic cell. Blood Rev.

[6] Chiorazzi N (2012). Implications of new prognostic markers in chronic lymphocytic leukemia. Hematol Am Soc Hematol Educ Program.

[7] Hamblin TJ (2007). Prognostic markers in chronic lymphocytic leukaemia. Best Pract Res Clin Haematol.

[8] Hamblin TJ, Davis Z, Gardiner A, Oscier DG, Stevenson FK (1999). Unmutated Ig V(H) genes are associated with a more aggressive form of chronic lymphocytic leukemia. Blood.

[9] Damle RN, Wasil T, Fais F, Ghiotto F, Valetto A, Allen SL, Buchbinder A, Budman D, Dittmar K, Kolitz J, Lichtman SM, Schulman P, Vinciguerra VP, Rai KR, Ferrarini M, Chiorazzi N (1999). Ig V gene mutation status and CD38 expression as novel prognostic indicators in chronic lymphocytic leukemia. Blood.

[10] Crespo M, Bosch F, Villamor N, Bellosillo B, Colomer D, Rozman M, Marce S, Lopez-Guillermo A, Campo E, Montserrat E (2003). ZAP-70 expression as a surrogate for immunoglobulin-variable-region mutations in chronic lymphocytic leukemia. N Engl J Med.

[11] Dohner H, Stilgenbauer S, Benner A, Leupolt E, Krober A, Bullinger L, Dohner K, Bentz M, Lichter P (2000). Genomic aberrations and survival in chronic lymphocytic leukemia. N Engl J Med.

[12] Chiorazzi N (2007). Cell proliferation and death: forgotten features of chronic lymphocytic leukemia B cells. Best Pract Res Clin Haematol.

[13] Messmer BT, Messmer D, Allen SL, Kolitz JE, Kudalkar P, Cesar D, Murphy EJ, Koduru P, Ferrarini M, Zupo S, Cutrona G, Damle RN, Wasil T, Rai KR, Hellerstein MK, Chiorazzi N (2005). In vivo measurements document the dynamic cellular kinetics of chronic lymphocytic leukemia B cells. J Clin Invest.

[14] Ajchenbaum F, Ando K, DeCaprio JA, Griffin JD (1993). Independent regulation of human D-type cyclin gene expression during G1 phase in primary human T lymphocytes. J Biol Chem.

[15] Parada Y, Banerji L, Glassford J, Lea NC, Collado M, Rivas C, Lewis JL, Gordon MY, Thomas NS, Lam EW (2001). BCR-ABL and interleukin 3 promote haematopoietic cell proliferation and survival through modulation of cyclin D2 and p27Kip1 expression. J Biol Chem.

[16] Solvason N, Wu WW, Parry D, Mahony D, Lam EW, Glassford J, Klaus GG, Sicinski P, Weinberg R, Liu YJ, Howard M, Lees E (2000). Cyclin D2 is essential for BCR-mediated proliferation and CD5 B cell development. Int Immunol.

[17] Wagner EF, Hleb M, Hanna N, Sharma S (1998). A pivotal role of cyclin D3 and cyclin-dependent kinase inhibitor p27 in the regulation of IL-2-, IL-4-, or IL-10-mediated human B cell proliferation. J Immunol.

[18] Boonen GJ, van Dijk AM, Verdonck LF, van Lier RA, Rijksen G, Medema RH (1999). CD28 induces cell cycle progression by IL-2-independent down-regulation of p27kip1 expression in human peripheral T lymphocytes. Eur J Immunol.

[19] Mohapatra S, Agrawal D, Pledger WJ (2001). p27Kip1 regulates T cell proliferation. J Biol Chem.

[20] Brunner MC, Chambers CA, Chan FK, Hanke J, Winoto A, Allison JP (1999). CTLA-4-mediated inhibition of early events of T cell proliferation. J Immunol.

[21] Greenwald RJ, Oosterwegel MA, van der Woude D, Kubal A, Mandelbrot DA, Boussiotis VA, Sharpe AH (2002). CTLA-4 regulates cell cycle progression during a primary immune response. Eur J Immunol.

[22] Kosmaczewska A, Ciszak L, Suwalska K, Wolowiec D, Frydecka I (2005). CTLA-4 overexpression in CD19+/CD5+ cells correlates with the level of cell cycle regulators and disease progression in B-CLL patients. Leukemia.

[23] Liang H, Nishioka Y, Reich CF, Pisetsky DS, Lipsky PE (1996). Activation of human B cells by phosphorothioate oligodeoxynucleotides. J Clin Invest.

[24] Decker T, Schneller F, Kronschnabl M, Dechow T, Lipford GB, Wagner H, Peschel C (2000). Immunostimulatory CpG-oligonucleotides induce functional high affinity IL-2 receptors on B-CLL cells: costimulation with IL-2 results in a highly immunogenic phenotype. Exp Hematol.

[25] Alegre ML, Shiels H, Thompson CB, Gajewski TF (1998). Expression and function of CTLA-4 in Th1 and Th2 cells. J Immunol.

[26] Linsley PS, Bradshaw J, Greene J, Peach R, Bennett KL, Mittler RS (1996). Intracellular trafficking of CTLA-4 and focal localization towards sites of TCR engagement. Immunity.

[27] Kuiper HM, Brouwer M, Linsley PS, van Lier RA (1995). Activated T cells can induce high levels of CTLA-4 expression on B cells. J Immunol.

[28] Lindsten T, Lee KP, Harris ES, Petryniak B, Craighead N, Reynolds PJ, Lombard DB, Freeman GJ, Nadler LM, Gray GS, Thompson CB, June CH (1993). Characterization of CTLA-4 structure and expression on human T cells. J Immunol.

[29] De TD, Reato G, Mauro F, Cignetti A, Ferrini S, Guarini A, Gobbi M, Grossi CE, Foa R (1999). IL4 production and increased CD30 expression by a unique CD8+ T-cell subset in B-cell chronic lymphocytic leukaemia. Br J Haematol.

[30] Mu X, Kay NE, Gosland MP, Jennings CD (1997). Analysis of blood T-cell cytokine expression in B-chronic lymphocytic leukaemia: evidence for increased levels of cytoplasmic IL-4 in resting and activated CD8 T cells. Br J Haematol.

[31] Rossmann ED, Lewin N, Jeddi-Tehrani M, Osterborg A, Mellstedt H (2002). Intracellular T cell cytokines in patients with B cell chronic lymphocytic leukaemia (B-CLL). Eur J Haematol.

[32] Douglas RS, Capocasale RJ, Lamb RJ, Nowell PC, Moore JS (1997). Chronic lymphocytic leukemia B cells are resistant to the apoptotic effects of transforming growth factor-beta. Blood.

[33] Trentin L, Zambello R, Agostini C, Siviero F, Adami F, Marcolongo R, Raimondi R, Chisesi T, Pizzolo G, Semenzato G (1993). Expression and functional role of tumor necrosis factor receptors on leukemic cells from patients with type B chronic lymphoproliferative disorders. Blood.

[34] Damle RN, Ghiotto F, Valetto A, Albesiano E, Fais F, Yan XJ, Sison CP, Allen SL, Kolitz J, Schulman P, Vinciguerra VP, Budde P, Frey J, Rai KR, Ferrarini M, Chiorazzi N (2002). B-cell chronic lymphocytic leukemia cells express a surface membrane phenotype of activated, antigen-experienced B lymphocytes. Blood.

[35] Kosmaczewska A, Ciszak L, Szteblich A, Laba A, Wojtowicz M, Wolowiec D, Frydecka I (2009). Is cyclin D2 a marker of B-cLL cell activation?. Oncol Res.

[36] Wolowiec D, Ciszak L, Kosmaczewska A, Bocko D, Teodorowska R, Frydecka I, Kuliczkowski K (2001). Cell cycle regulatory proteins and apoptosis in B-cell chronic lymphocytic leukemia. Haematologica.

[37] Suwalska K, Pawlak E, Karabon L, Tomkiewicz A, Dobosz T, Urbaniak-Kujda D, Kuliczkowski K, Wolowiec D, Jedynak A, Frydecka I (2008). Association studies of CTLA-4, CD28, and ICOS gene polymorphisms with B-cell chronic lymphocytic leukemia in the Polish population. Hum Immunol.

[38] Joshi AD, Hegde GV, Dickinson JD, Mittal AK, Lynch JC, Eudy JD, Armitage JO, Bierman PJ, Bociek RG, Devetten MP, Vose JM, Joshi SS (2007). ATM, CTLA4, MNDA, and HEM1 in high versus low CD38 expressing B-cell chronic lymphocytic leukemia. Clin Cancer Res.

[39] Decker T, Hipp S, Kreitman RJ, Pastan I, Peschel C, Licht T (2002). Sensitization of B-cell chronic lymphocytic leukemia cells to recombinant immunotoxin by immunostimulatory phosphorothioate oligodeoxynucleotides. Blood.

[40] Bogner C, Schneller F, Hipp S, Ringshausen I, Peschel C, Decker T (2003). Cycling B-CLL cells are highly susceptible to inhibition of the proteasome: involvement of p27, early D-type cyclins, Bax, and caspase-dependent and -independent pathways. Exp Hematol.

[41] Decker T, Schneller F, Hipp S, Miething C, Jahn T, Duyster J, Peschel C (2002). Cell cycle progression of chronic lymphocytic leukemia cells is controlled by cyclin D2, cyclin D3, cyclin-dependent kinase (cdk) 4 and the cdk inhibitor p27. Leukemia.

[42] Decker T, Hipp S, Ringshausen I, Bogner C, Oelsner M, Schneller F, Peschel C (2003). Rapamycin-induced G1 arrest in cycling B-CLL cells is associated with reduced expression of cyclin D3, cyclin E, cyclin A, and survivin. Blood.

[43] Longo PG, Laurenti L, Gobessi S, Petlickovski A, Pelosi M, Chiusolo P, Sica S, Leone G, Efremov DG (2007). The Akt signaling pathway determines the different proliferative capacity of chronic lymphocytic leukemia B-cells from patients with progressive and stable disease. Leukemia.

[44] Erlanson M, Landberg G (2001). Prognostic implications of p27 and cyclin E protein contents in malignant lymphomas. Leuk Lymphoma.

[45] Vrhovac R, Delmer A, Tang R, Marie JP, Zittoun R, Ajchenbaum-Cymbalista F (1998). Prognostic significance of the cell cycle inhibitor p27Kip1 in chronic B-cell lymphocytic leukemia. Blood.

[46] Wolowiec D, Wojtowicz M, Ciszak L, Kosmaczewska A, Frydecka I, Potoczek S, Urbaniak-Kujda D, Kapelko-Slowik K, Kuliczkowski K (2009). High intracellular content of cyclin-dependent kinase inhibitor p27(Kip1) in early- and intermediate stage B-cell chronic lymphocytic leukemia lymphocytes predicts rapid progression of the disease. Eur J Haematol.

[47] Delmer A, Ajchenbaum-Cymbalista F, Tang R, Ramond S, Faussat AM, Marie JP, Zittoun R (1995). Overexpression of cyclin D2 in chronic B-cell malignancies. Blood.

[48] Chiles TC (2004). Regulation and function of cyclin D2 in B lymphocyte subsets. J Immunol.

[49] Liang J, Slingerland JM (2003). Multiple roles of the PI3 K/PKB (Akt) pathway in cell cycle progression. Cell Cycle.

[50] Schneider H, Valk E, Leung R, Rudd CE (2008). CTLA-4 activation of phosphatidylinositol 3-kinase (PI 3-K) and protein kinase B (PKB/AKT) sustains T-cell anergy without cell death. PLoS ONE.

